# Non-invasive cardiac pacing with image-guided focused ultrasound

**DOI:** 10.1038/srep36534

**Published:** 2016-11-09

**Authors:** Fabrice Marquet, Pierre Bour, Fanny Vaillant, Sana Amraoui, Rémi Dubois, Philippe Ritter, Michel Haïssaguerre, Mélèze Hocini, Olivier Bernus, Bruno Quesson

**Affiliations:** 1 IHU Liryc, Electrophysiology and Heart Modeling Institute, Fondation Bordeaux Université, F-33600 Pessac- Bordeaux, France; 2 Univ. Bordeaux, Centre de recherche Cardio-Thoracique de Bordeaux, U1045, F-33000, Bordeaux, France; 3 INSERM, Centre de recherche Cardio-Thoracique de Bordeaux, U1045, F-33000 Bordeaux, France; 4 Image-Guided Therapy SA, F-33600 Pessac, France; 5 Bordeaux University Hospital (CHU), Electrophysiology and Ablation Unit, F-33600 Pessac, France; 6 Bordeaux University Hospital (CHU), Electrophysiology and Cardiac Stimulation Unit, F-33600 Pessac, France

## Abstract

Currently, no non-invasive cardiac pacing device acceptable for prolonged use in conscious patients exists. High Intensity Focused Ultrasound (HIFU) can be used to perform remote pacing using reversibility of electromechanical coupling of cardiomyocytes. Here we described an extracorporeal cardiac stimulation device and study its efficacy and safety. We conducted experiments *ex vivo* and *in vivo* in a large animal model (pig) to evaluate clinical potential of such a technique. The stimulation threshold was determined in 10 different *ex vivo* hearts and different clinically relevant electrical effects such as consecutive stimulations of different heart chambers with a single ultrasonic probe, continuous pacing or the inducibility of ventricular tachycardia were shown. Using ultrasonic contrast agent, consistent cardiac stimulation was achievable *in vivo* for up to 1 hour sessions in 4 different animals. No damage was observed in inversion-recovery MR sequences performed *in vivo* in the 4 animals. Histological analysis revealed no differences between stimulated and control regions, for all *ex vivo* and *in vivo* cases.

The predominant technique for temporary cardiac pacing used in the clinical setting is invasive consisting in the insertion of intracardiac catheters. Although pacing with leads has been successfully used clinically for almost 60 years, these catheters are associated with costs (single-use devices, need of a sterile environment) and inherent risks^1^,^2^,^3^,^4^ from the nature of interventional procedure. Also long-term complications with leads are not negligible, therefore ways to stimulate the heart without leads are intensively looked for. The unique non-invasive method is the transcutaneous transthoracic electrical stimulation. This technique is frequently not accepted by patients because of the discomfort of the subcutaneous stimulation of the thoracic musculature as effective cardiac pacing requires high intensity pulses most of the time^5^.

In this manuscript we present a purely extracorporeal approach to perform contactless cardiac stimulation using High Intensity Focused Ultrasound (HIFU). Although cardiac contractions in sinus rhythm result from electrical activation of cardiac myocytes, local mechanical stress applied to cardiac tissues can also induce cardiac depolarization^6^. Local mechanical stress in internal organs can be achieved by concentrating ultrasonic waves emitted by an extracorporeal transducer. The ability of ultrasound to directly cause premature ventricular contractions (PVC) was first described by Harvey in 1929^7^. More recently, the effect of ultrasound on cardiac tissue has been studied in small animals^8^,^9^,^10^,^11^,^12^,^13^ in experiments mainly designed to determine safety guidelines for echocardiography. Ultrasound-induced cardiac arrhythmias have been reported as a side effect of lithotripsy^14^. This therapy uses shock waves to destroy kidney stones and has to be synchronized to the electrocardiogram to reduce the induction of cardiac arrhythmias^15^. To the best of our knowledge, proof of concept of controlled HIFU stimulation for therapeutic purposes has only been performed on small animals^16^,^17^ with limited pacing success (around 15%). Clinical applicability is also difficult to assess because small animal models artificially circumvent technical difficulties such as the limited acoustic window (acoustic reflection/absorption by the rib cage and the lungs) or respiratory and cardiac motions.

In the present study, we demonstrated proof of feasibility of non-invasive cardiac stimulation with focused ultrasound in a large animal model *ex vivo* and *in vivo.* Specifically, we aimed to develop a pacing device offering the following conditions:

- purely extracorporeal application,
- precise targeting (≤5 mm uncertainty) of predetermined region(s) within the heart,
- ECG gating to allow for precise timing of the contraction of the targeted cardiac chamber(s),
- reliable stimulation response and no associated tissue alteration,
- consecutive pacing of different heart chambers with an adjustable delay, and
- real-time electrical and hemodynamic assessment.

In the first phase of this proof of concept, we determined the acoustic threshold required to achieve reliable stimulation in the atrium and ventricle of ten *ex vivo* pig hearts as a function of the ultrasound pulse duration. We also investigated various types of ultrasound-induced electrical effects, such as consecutive stimulations of different heart chambers with the same probe using ultrasound beam steering capabilities, continuous ventricular pacing or induction of ventricular tachycardia with a single ultrasound pulse. In the second phase, we performed *in vivo* proof of feasibility in four pigs, assessing the safety of the procedure using Magnetic Resonance Imaging (MRI), gross examination and histology.

## Results

### *Ex vivo* acoustic stimulation threshold determination

**Figure 1:**
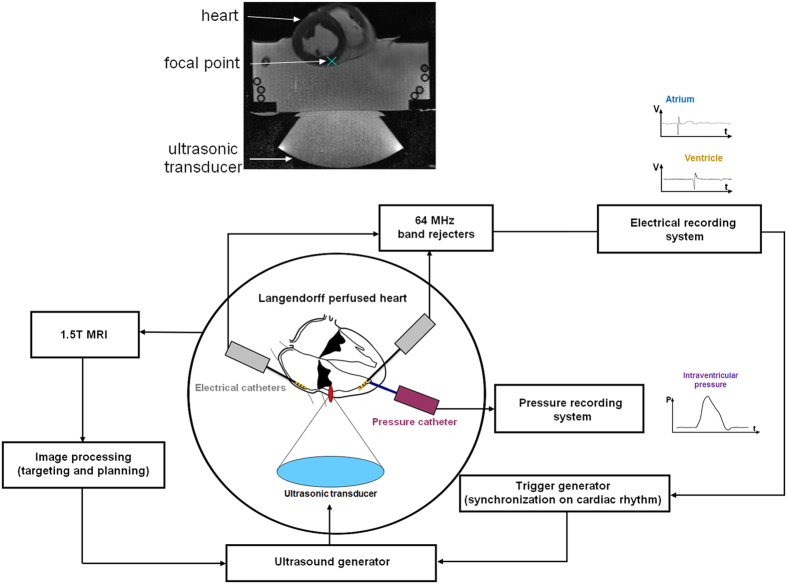
Schematic of the *ex vivo* experimental setup and corresponding MR image. A beating pig heart (Langendorff perfusion) is set in a tank filled with a mix of Tyrode buffer and autologous blood. The ultrasonic probe (256 elements phased-array operating at 1 MHz) is positioned underneath and can sonicate the different heart cavities through the acoustic window. This setup is placed inside a 1.5T MR scan for image guidance. Electrical and intraventricular recordings are recorded in the different heart cavities from MRI compatible catheters and the ultrasound pulses are synchronized on the heart cycle with adjustable delays.

**Figure 2:**
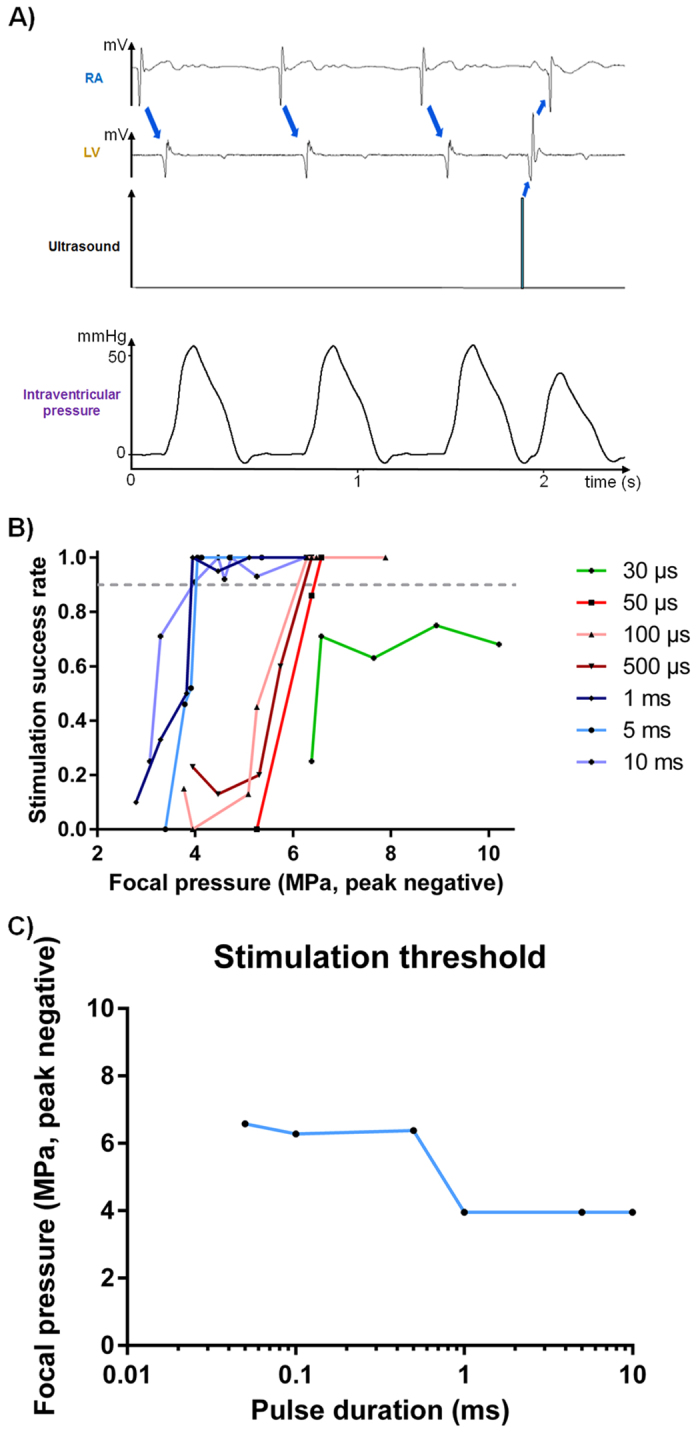
(**A**) Example of ultrasound-induced premature ventricular contraction. Figure reports electrocardiograms for RA and LV, focus ultrasound signal and left intraventricular pressure. After three sinus rhythm beats, an ultrasonic pulse is sent after the absolute refractory period of the ventricle. A ventricular depolarization can be observed. Actual ultrasound-induced heart contraction was confirmed by intraventricular pressure measurements recorded simultaneously. (**B**) Stimulation success rate for different ultrasound pulse durations as a function of the acoustic pressure at focus. The grey dashed line shows the 90% success rate threshold chosen. (**C**) Stimulation threshold ensuring at least 90% stimulation in peak negative pressure reported as a function of the pulse duration.

Figure 1 illustrates the *ex vivo* experimental setup used. It combines an isolated heart perfused in Langendorff mode, an MR-compatible 256-element phased array HIFU transducer (1 MHz central frequency), and measurement of local cardiac electrograms from different heart chambers and intraventricular pressure measurements. Local electrograms are used to synchronize the ultrasonic emission on the heart cycle and intraventricular pressure recordings are used to confirm mechanical contraction of the heart. Using the setup described in Fig. 1 (see Material and Methods section) ultrasound-induced extrasystole can be achieved. An example can be seen in Fig. 2A. On these recordings, three normal heart contractions in sinus rhythm are displayed before an ultrasonic stimulation attempt was made (5 MPa peak negative, 5 ms pulse length). This mechanical stimulus induced a ventricular depolarization followed by a retrograde conduction in the atrium, resulting in a PVC (peak-to-peak time delay of 580 ms compared to 650 ms in sinus rhythm) and lower maximum pressure (42 mmHg compared to 54 mmHg in sinus rhythm).

The results of the 756 stimulation sites performed in the right atrium (RA, 83 sonications), and the left and right ventricles (431 and 242 sonications respectively) in 10 *ex vivo* beating hearts from pigs are depicted in Fig. 2B. The success rate of stimulation for different pulse durations is plotted as a function of the acoustic pressure at the focus (peak negative). For each HIFU pulse duration tested ranging from 30 μs to 10 ms, the success of stimulation increases with the acoustic pressure at focus. To determine the stimulation pressure threshold, the pressure values associated with a stimulation success rate of at least 90% are reported as a function of pulse duration (Fig. 2C). Two different pressure thresholds are highlighted: one around 4 MPa peak negative for HIFU pulse durations above 1 ms and one around 6 MPa peak negative for HIFU pulses ranging from 50 μs to 1 ms. For HIFU pulse durations shorter than 50 μs, the 90% stimulation threshold was not reached in this study (10 MPa peak negative, maximal success rate around 70%).

### Various ultrasound-induced electrical effects

**Figure 3:**
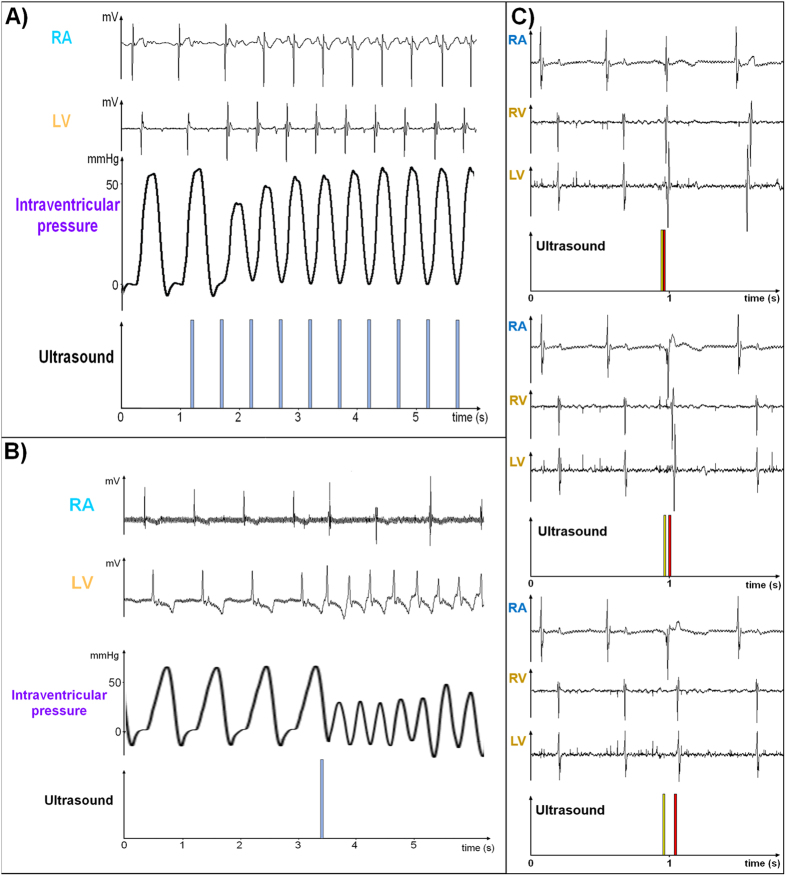
(**A**) Electrical and hemodynamic recordings of continuous ultrasonic pacing of the heart at 120 bpm (sinus rhythm: 100 bpm). (**B**) Electrical and hemodynamic recordings of ultrasound-induced ventricular tachycardia (165 bpm, sinus rhythm 85 bpm) after triggering the acoustic pulse during the relative refractory period. (**C**) Example of atrioventricular stimulation with a single ultrasonic probe. Phased array transducer enables consecutive stimulation of the RA (yellow pulse) and the RV (red pulse) with a programmed delay (from top to bottom: 0 ms, 40 ms and 120 ms interpulse delay, respectively).

An example of HIFU continuous pacing is depicted in Fig. 3A. Spontaneous rate was around 100 bpm and the heart was paced at 120 bpm. As ECG gating was not used, the first pulse did not result in capture of the ventricles as it fell during the absolute refractory period after the spontaneous ventricular depolarization. Ventricular pacing was efficient from the second pulse. The actual mechanical contraction was again confirmed by the intraventricular pressure measurement. The first two rapid paced beats exhibited lower systolic pressure, then circulation progressively adjusted to the faster pace with restoration of the previous pressure after 6–7 beats.

It has already been demonstrated that ventricular tachycardia can be induced by stimulation during the ventricular vulnerable refractory period^18^, a phenomenon also observed in our ultrasonic experiments as illustrated in Fig. 3B. Before stimulation, sinus rhythm was around 85 bpm. Ultrasonic stimulation induced a ventricular tachycardia up to 165 bpm (2^nd^ trace from the top in Fig. 3B) with atrio-ventricular dissociation (top trace in Fig. 3B) resulting in impairment of hemodynamics (3^rd^ trace from top in Fig. 3B).

Consecutive stimulations of the RA and the RV with the desired delay can be achieved using a single ultrasonic emitter (example in Fig. 3C), exploiting the beam steering capability of our phased-array ultrasound transducer. The yellow HIFU pulse was targeted in the RA while the red HIFU pulse was targeted in the RV. The interpulse delay was set to 0 ms, 40 ms and 120 ms (from top to bottom in Fig. 3C), demonstrating the ability to adjust the atrioventricular delay. Direct stimulation of the RA and RV as well as electrical propagation from the RV to the LV can be seen in all the electrical traces in Fig. 3C.

### *In vivo* proof of feasibility of HIFU stimulation

**Figure 4:**
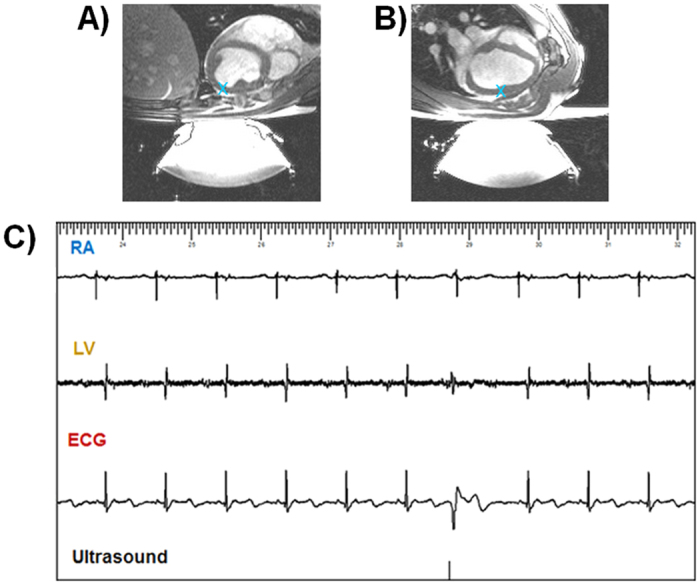
*In vivo* proof of concept of non-invasive cardiac stimulation. (**A**,**B**) Sagittal and transverse MR images of the anesthetized pig (blue cross depicts targeted region). Using our current setup, adjunction of ultrasound contrast agent was necessary to consistently induce PVCs *in vivo*. (**C**) shows an example of such stimulation in the second animal with complete electrical recordings in both RA and LV as well as surface ECG. The acoustic pulse induced a premature ventricular depolarization observed as a premature QRS complex on the ECG with a different morphology as compared to the conducted QRS.

The minimal stimulation threshold of 4 MPa negative pressure at the focus (as determined from *ex vivo* experiments) could not be reached with our current *in vivo* setup. The maximal peak negative pressure was estimated to be around 2 MPa *in situ*, due to the limited acoustic window (see Materials and Methods section). At this pressure level, stimulation of the LV was observed but with an insufficient success rate. To overcome this limitation and demonstrate *in vivo* feasibility, ultrasound contrast agents were injected intravenously to enhance HIFU mechanical effects on tissue, hence decreasing the stimulation threshold. Figure 4 shows examples of *in vivo* data. Complete electrical recordings in both the RA and the LV using the same MR-compatible catheters as well as surface ECG were available. Using this protocol, consistent cardiac stimulation was achievable for up to 1 hour sessions in 4 different animals.

**Figure 5:**
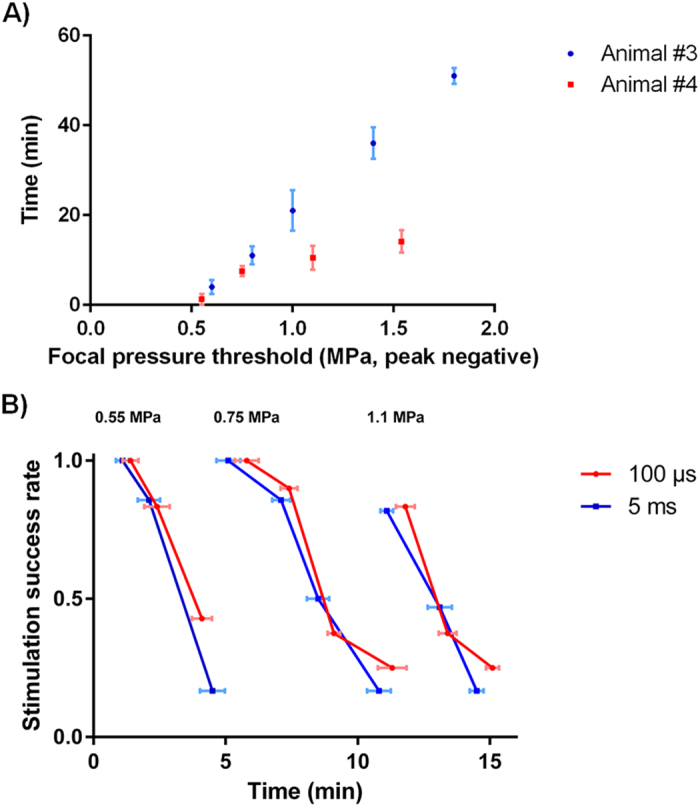
(**A**) Time when it was necessary to increase the acoustic amplitude to induce consistent cardiac depolarization as a function of the peak negative pressure *in situ* due to ultrasound contrast agent clearance. (**B**) Evolution of the stimulation success rate according to time of application for short and long pulses (100 μs and 5 ms respectively) at different pressure levels (0.55 MPa, 0.75 MPa and 1.1 MPa peak negative).

For the last two animals, a preliminary investigation of the effects of ultrasound contrast agent concentration on stimulation threshold was performed. The lowest HIFU pressure to induce cardiac depolarization was determined using a pulse duration of 5 ms. 5 peak negative pressure steps were defined for animal #3 (0.6, 0.8, 1.0, 1.4 and 1.9 MPa after taking the total attenuation into account) and 4 for animal #4 (0.55, 0.75, 1.1 and 1.6 MPa after taking total attenuation into account). The results are depicted in Fig. 5A. Differences in *in situ* pressure levels can be explained by slightly different animal orientation and anatomy leaving a different surface of the acoustic beam intercepted by the ribs and the lungs (see Materials and Methods section).

A preliminary comparison of short and long pulses (100 μs and 5 ms) was also performed on the last animal. Three different pressure levels were applied across time to check whether pulse duration had an effect on ultrasonic stimulation. Results are shown in Fig. 5B. As opposed to the *ex vivo* case without adjunction of ultrasonic contrast agents, the pulse duration did not have any effect on the stimulation threshold in the presence of microbubbles.

### Preliminary safety assessment

**Figure 6:**
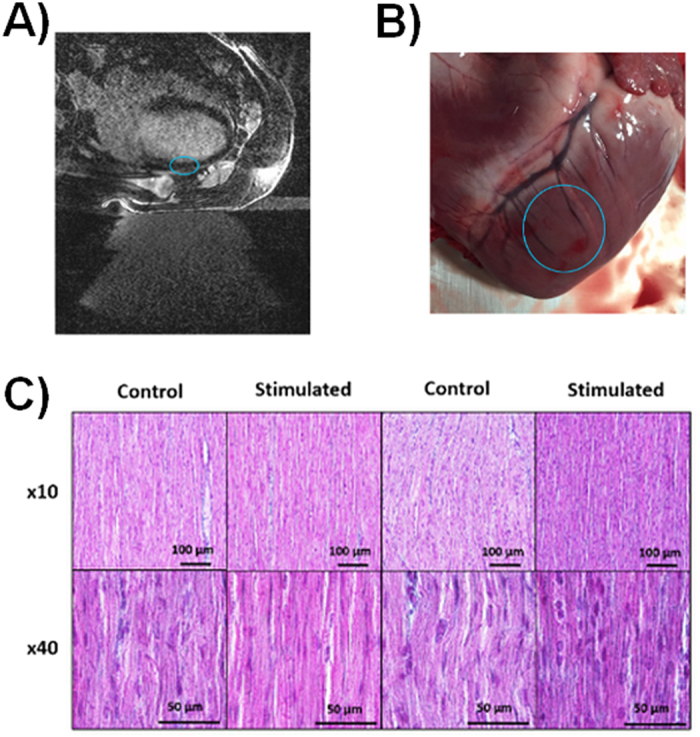
Examples of safety reports from the 4 *in vivo* cases. (**A**) 3D Inversion-recovery MR sequences did not reveal any contrast agent enhancement in the myocardium meaning that no scar or edema were detectable (blue circle depicts sonicated region). (**B**) Gross examination did not reveal any particular damage in the sonicated region of any animal (blue circle depicts sonicated region). (**C**) Histology (Masson’s staining) slices analysis did not show noticeable differences between control and sonicated regions.

Preliminary safety assessment was performed for all the 10 *ex vivo* and 4 *in vivo* cases. Figure 6 shows examples of safety assessment for one of the four *in vivo* cases. No damage was observed in inversion-recovery MR sequences performed *in vivo* in the 4 animals (Fig. 6A). No signal increase can be seen in the myocardium in the delayed-enhancement MR images that would indicate irreversible injury. Gross pathology (Fig. 6B) and Masson’s staining (Fig. 6C) revealed no differences between stimulated and control regions, for all the cases for which samples were collected (4 *ex vivo* and 4 *in vivo*). No evidence of coagulative necrosis, hemorrhage or interstitial edema was found in the samples. For the *in vivo* cases, no damage was noted at visual inspection on the skin, esophagus, lungs and rib cage. Therefore, no further histological analysis was conducted on these organs.

## Discussion

The need for a less intrusive approach has been highlighted with the recent development of leadless pacing^19^,^20^,^21^. This promising technique offers improvements in both reduction of invasiveness and improved cost-effectiveness at the expense of implant risks due to large diameter device (perforation) and issues with replacement and retrieval of the device (dislodgement). However this approach remains mini-invasive. To the best of our knowledge, this study is the first *ex vivo* and *in vivo* proof of feasibility of controlled non-invasive ultrasound-based cardiac stimulation in large animals. The *ex vivo* characterization demonstrated the potential of this technique in an environment where acoustic parameters were well-controlled and quantitatively determined the stimulation threshold as a function of ultrasound pulse duration and amplitude. The *in vivo* proof of feasibility performed in large animals showed that this novel technology offers good prospects for clinical developments.

In the clinical setting, non-invasive ultrasound cardiac pacing could be useful in various conditions:

- For temporary pacing in patients suffering from bradycardia or any clinical condition with risks of cardiac asystole. As the ultrasound beam can be focused on very specific cardiac zones, temporary pacing could be applied to the atrium in case of sinus node disease, to the ventricle in case of AV block or even at both sites to restore AV synchronization in patient whom hemodynamic support must be achieved.

- In the context of electrophysiology for terminating tachyarrhythmia that compromise hemodynamics, or for examining the inducibility of tachyarrhythmia.

- For hemodynamic studies in order to analyze the possible benefits of atrial chamber pacing in obstructive cardiomyopathy or of optimal AV delay programming in complete AV block patients. This technique could also be used in CRT, to optimize the RV and LV leads placement and assessing efficacy of programming settings (AV delay and VV interval).

Pressure values needed to depolarize the myocardium were in the same range as those reported in small animals^8^,^11^. The shape of the stimulation threshold curve for the acoustic stimulation at 1 MHz displays two well identified pressure thresholds for pulse durations shorter or longer than 1 ms. This suggests the existence of two distinct mechanical stimulation mechanisms, *e.g.* cavitation^22^, radiation force^23^ or another process. Cavitation has already been suggested as one of the driving mechanisms in small animal studies, as adjunction of ultrasound contrast agents decreased the stimulation threshold^12^,^13^. Our study supported these findings. Acoustic backscattered signals from the focus have been recorded during both *ex vivo* and *in vivo* experiments. Inertial cavitation was found to be significant above 2.2 MPa peak negative *ex vivo* and 0.7 MPa peak negative *in vivo* in the presence of ultrasound contrast agent (see supplemental data). However, real-time monitoring of both stable and inertial cavitation doses need to be further investigated. Those metrics have been linked to treatment efficacy and safety for the disruption of the blood-brain barrier using focused ultrasound^24^,^25^.

In the presence of an ultrasound contrast agent, no differences in minimal pressure to induce cardiac depolarization were found *in vivo* between short and long pulses (100 μs and 5 ms) suggesting that cavitation may be the preferred mechanism under these experimental conditions. Long lasting stimulation was possible *ex vivo* however in its current configuration the transducer design is suboptimal for this application *in vivo* and was unable to generate sufficient focal pressure due to attenuation of ultrasonic waves by the rib cage. As such, consistent cardiac depolarization could not be achieved without the adjunction of ultrasound contrast agents. The aperture of the ultrasound probe has to be adapted to the small acoustic window due to the presence of ribs or lungs and both respiratory and cardiac motions have to be taken into account. Real-time target tracking and adaptive beam-steering^26^ might improve the efficacy of non-invasive cardiac stimulation without the adjunction of ultrasound contrast agent. This requires further development of dedicated hardware and software to perform efficient non-invasive cardiac pacing *in vivo* without the adjunction of ultrasound contrast agent.

*In vivo* and *ex vivo* imaging and histology preliminary safety studies did not show any differences between sonicated and control tissues. These encouraging results show that acute stimulation during hour-long sessions did not cause any detectable thermal and mechanical damage under the experimental parameters used. Ongoing work includes a complete safety study *in vivo* using various stimulation protocols. This investigation coupled with the aforementioned real-time cavitation monitoring study is crucial to judge the clinical potential of this technique. This is motivated by reports of macroscopic lesions using ultrasound contrast agent and focused ultrasound targeting the myocardium in rats under extreme exposure conditions (respectively 15.9 MPa and up to 8 MPa *in situ* peak negative pressure, both using ultrasound contrast agent)^12^,^27^. Eventually a complete preclinical chronic safety investigation will be performed. Such a study could be performed collecting blood samples during non-invasive stimulation sessions and biomarkers of cardiac injury such as myoglobin, lactate dehydrogenase, creatine kinase and troponin could be looked for. These biomarkers have been proven to correlate well with cardiac injury in different large animal models^28^,^29^.

The present study also shows a number of limitations. The success rate threshold for stimulation was arbitrarily set to 90% to account for the few occurrences of false negatives (see Fig. 2B). Although arbitrary, the resulting pressure thresholds for short and long pulses are relevant as modification of this threshold will not result in drastic changes in the minimal pressure needed to induce a cardiac depolarization. These false negatives might be caused by the experimental conditions. Firstly, swinging motion of the *ex vivo* heart on the polyurethane membrane might have occurred during its contraction. Also, the heart was not paced and was beating in sinus rhythm. Therefore when stimulating different regions of the ventricles, some sonications could have occurred during the absolute refractory period. *Ex vivo* assessment of the ultrasound contrast effect on the stimulation threshold was complicated due to the Langendorff perfusion (comprising pump and oxygenator that would not allow microbubbles to be stable for a sufficient time). However this study demonstrates the potential of such an ultrasound-based non-invasive cardiac stimulation in large animals that may provide a new approach to regulate cardiac function.

Last but not least, MRI was used during this study as the main imaging modality for targeting, planning and safety assessment. Other simpler imaging modalities could be used such as ultrasound imaging for real-time HIFU guidance. This other imaging modality might also be used for hemodynamic assessment and coupled with extracorporeal ECG for a fully non-invasive procedure.

## Methods

### Chemicals

The chemicals and biological products were purchased from Sigma Aldrich.

### Animal preparation

The protocol was approved by the local Animal Research Ethics Committee (Comité d’Ethique en Expérimentation Animale de Bordeaux - CEEA50) and all experiments were performed in accordance with the approved guidelines. Pigs (Large White x Landrace, ~40 kg, 10 animals for *ex vivo* validation, 4 animals for *in vivo* proof of concept) were premedicated with ketamine (20 mg.kg^−1^) and acepromazine (1 mg.kg^−1^) injected IM. Induction of anesthesia was realized with intravenous bolus of ketamine (15 mg.kg^−1^) and midazolam (1.5 mg.kg^−1^). After induction of anesthesia, animals were intubated and ventilated, and received an injection of heparin (2.5 mg.kg^−1^). Anesthesia was maintained with ketamine and midazolam (40 mg.kg^−1^.h^−1^ and 2 mg.kg^−1^.h^−1^ respectively). During the *in vivo* experiments the animals were injected with ultrasound contrast agents using SonoVue (Bracco, Italy), mean terminal half-life: 12 min, range from 2 min to 33 min. Two consecutive 0.1 mL.kg^−1^ boli intravenous injection were performed in each animal.

### Heart extraction and surgery preparation

The thorax was opened and blood from each animal was collected (~3 L) via an 8Fr introducer inserted in the right jugular vein. Vascular filling was performed using Voluven IV infusion at a pace corresponding to the debit of the blood collected via the femoral vein. Cardiac arrest was induced *in vivo* by crossclamping of the ascending aorta and direct injection in the aortic root of 1 L of cold (4 °C) cardioplegic Celsior^®^ (Genzyme, Saint-German-en-Laye, France) solution, following by a rapid excision and immersion in a cold 0.9% saline solution. The extraction protocol was based on standard clinical extraction^30^.

### Isolated heart perfusion in Langendorff mode

The aorta and the pulmonary artery were cannulated. The pulmonary veins and the inferior and superior vena cava were sutured to limit leaks. The entire coronary flow goes into the coronary sinus, the right atrium and ventricle, and was collected through the pulmonary artery. The hearts were then placed onto a polyurethane membrane (transparent to ultrasound) in a plexiglas thermoregulated tank and reperfused in the Langendorff mode at 60–70 mmHg of coronary perfusion pressure. When necessary, hearts were defibrillated at 10–30 J. This set-up offers loaded ventricles with the perfusion medium from the coronary vascular bed, resulting in an isovolumetric contraction. Perfusion of the heart was realized with autologous blood diluted with a Tyrode buffer (v/v: 1/3, gassed with O_2_/CO_2_ 95/5%, pH 7.4) maintained at 38 °C. Tyrode buffer contains physiological concentrations of hormones (8 nM insulin, 5 nM epinephrine) and metabolic substrates (16 mM glucose, 0.5 mM pyruvate, 1 mM lactate)^31^.

### Intraventricular pressure measurements

A homemade intraventricular pressure catheter was inserted into the left ventricle via the apex and connected to a fluid-filled piezoelectric pressure transducer for continuous monitoring of left ventricular pressure (EMKA-IOX2 data acquisition system, EMKA Technologies, VA, USA).

### Electrical measurements

Local cardiac electrograms (bipolar measurements) were continuously recorded by three MR-compatible pacemaker leads (CapSureFix MRI Model 5086, Medtronic, MN, USA) inserted into the right ventricle, the left ventricle and the right atrium and connected to a clinical electrophysiology recording system (Bard Inc., NJ, USA) through dedicated filters specifically designed for the MR environment (band rejecters at 64 MHz which is the proton resonant frequency at 1.5T). A custom trigger generator was manufactured in-house which can be set to synchronize the ultrasonic emissions and the desired delay to the electric signal of any heart cavities.

### MR-guided HIFU

Experiments were performed with an MR-guided HIFU platform combining a 1.5T MRI (Siemens Avanto, Germany) and a focused ultrasound device (Image Guided Therapy, France, 256 elements, 13/13 cm aperture/focal, operating at 1 MHz). MR images were recorded using a balanced steady-state free precession sequence (TE/TR/FA/BW = 1.36 ms/493 ms/80°/1149 Hz.pixel^−1^, spatial resolution 1 × 1 mm^2^, slice thickness 3 mm, 256 × 256, 40 slices, 3 stacks acquired in transverse, sagittal and coronal orientations) to select the location of the stimulation site and to adjust beam focusing characteristics (mechanical positioning and electronic beam steering). The HIFU transducer was calibrated using two needle hydrophones (0.075 mm at pressure below 2 MPa peak negative and 0.5 mm diameter at pressure above 2 MPa peak negative, Precision Acoustics, Dorchester, UK) in a deionized and degassed water tank. The focus was measured at −6 dB to be 1.8 mm wide in the focal plane and 12.6 mm wide along the focal axis. *In situ* focal pressures were determined taking into account absorption in the diluted blood and heart tissue^32^ (respectively 0.20 dB.MHz^−1^.cm^−1^ and 0.52 dB.MHz^−1^.cm^−1^) and acoustic loss due to electronic deflection applied during the experiment to steer the beam away from the transducer natural focus (attenuation of 2.50 dB.cm^−1^ laterally and 0.45 dB.cm^−1^ axially, measured by hydrophone in a water tank). For determining *in vivo* pressure at focus, an additional attenuation was applied to take into account for reflected acoustic energy by the ribs and the lungs (percentage of the acoustic beam surface intercepted by the ribs and the lungs estimated on anatomical MR images) as well as attenuation induced by the fat layer^32^ (0.63 dB.MHz^−1^.cm^−1^).

### *Ex vivo* and *in vivo* stimulation

*Ex vivo* acoustic stimulation threshold was determined performing 756 sonications in the right atrium (83 sonications), the left (431 sonications) and the right ventricles (242 sonications) in 10 *ex vivo* beating hearts from pigs. *Ex vivo* continuous pacing was performed 11 times in 3 different pig hearts. Ventricular tachycardia was induced 6 times in 2 different pig hearts. Multisite stimulation was performed 15 times in 2 different hearts.

*In vivo* non-invasive stimulation proof of concept was shown performing 314 sonications in 4 anesthetized pigs including 42 sonications without ultrasound contrast agent in the first two animals. Correlation of cardiac susceptibility to stimulation with ultrasound contrast agent pharmacokinetic was performed in the last two animals (total of 84 sonications). Preliminary comparison of short and long ultrasonic pulses was performed in the last animal (total of 74 sonications).

### Data analysis

Ultrasound induced depolarization were identified by the following chain of events: ultrasound trigger representing the emission of the ultrasonic pulse, depolarization seen in the electrical recordings and mechanical contraction seen in the pressure recordings. To determine the stimulation thresholds, different values of stimulation acoustic amplitudes and durations were tested. Acoustic amplitudes ranged from 1.3 MPa to 10 MPa peak negative, and pulses duration ranged from 30 μs to 10 ms. For each amplitude-duration set, the success rate of inducing a premature ventricular contraction was measured. The stimulation was considered reliable when at least 90% success was observed for each amplitude-duration set, defining a stimulation threshold.

### Safety assessment

At the end of each *in vivo* experiment, a navigated delayed inversion-recovery 3D Flash sequence was performed (TE/TR/TI/FA/BW = 3.93 ms/714 ms/320 ms/13°/130 Hz.pixel^−1^, spatial resolution 0.5 × 0.5 mm^2^, slice thickness 2.5 mm, 576 × 576, 52 slices). The animals were injected with 0.2 mmol.kg^−1^ gadoterate meglumine (Gd-DOTA, Dotarem^®^, Guerbet, Roissy, France) and scanned 15 minutes post injection.

Gross examination of each heart was performed after the heart excision. The heart samples were then cut in small parts surrounding the sonicated areas. Control regions (absence of sonication) were also collected for comparison. Each sample was rapidly put in a 4% formaldehyde solution for a week. Samples were then dehydrated and included in paraffin. 8 μm thickness slices were cut using a microtome (RM2255, Leica, Germany). Coloration was performed using Masson’s trichrome staining protocol. One of every twenty slices was stained giving approximately a 160 μm distance between two analyzed slices (sometimes the exact slice was not cut properly, in that case the following one was used). Histological analysis were performed using Eclipse 80i microscope, a DS-QilMc camera and a DS-U3 digitizer (all from Nikon, Japan) to assess acute damages screening from acoustic stimulation. Tissue samples of stimulated heart (N = 40, stimulated at peak negative pressure below 5 MPa *ex vivo* and 2 MPa *in vivo* with contrast agents, each sample was stimulated at least 10 times) as well as control regions (N = 24) were collected in 4 *ex vivo* and 4 *in vivo* hearts. For the *in vivo* cases, the skin included into the beam path was observed to identify potential burns. Esophagus and lungs were extracted, examined and dissected (parallel slices of 3–5 mm for the lungs, cut in the long axis for the esophagus to observe the external and internal sides) for observing the potential presence of a lesion. In case a lesion could be identified, samples of the tissue in the affected and healthy areas were selected for histological analysis using the same protocol as for the heart. Finally, the internal face of the rib cage in the sonicated area was also observed to account for potential lesions.

## Additional Information

**How to cite this article**: Marquet, F. *et al.* Non-invasive cardiac pacing with image-guided focused ultrasound. *Sci. Rep.* **6**, 36534; doi: 10.1038/srep36534 (2016).

**Publisher’s note**: Springer Nature remains neutral with regard to jurisdictional claims in published maps and institutional affiliations.

## Supplementary Material

Supplementary Information
