# Supplementary material for: Non-invasive cardiac pacing with image-guided focused ultrasound
Source: Sci Rep. 2016 Nov 9;6:36534. doi: 10.1038/srep36534 (PMC5101517; doi:10.1038/srep36534)
Supplement: Supplementary Information [file srep36534-s1.pdf]

Non-invasive cardiac pacing with image-guided focused ultrasound.

Fabrice Marquet, Pierre Bour, Fanny Vaillant, Sana Amraoui, Rémi Dubois, Philippe Ritter, Michel Haïssaguerre, Mélèze Hocini, Olivier Bernus, Bruno Quesson

SUPPLEMENTARY DATA

Passive Cavitation Detection (PCD)

A single-element PCD (Y133_MR, 10kHz – 20 MHz bandwidth, 5 mm active diameter, unfocused MR-compatible hydrophone, Sonic Concepts, WA, USA) was connected to a digitizer (Oscar, 12-bit, 100MHz sampling frequency, Gage Applied Technologies, DynamicSignal LLC, IL, USA) through a 20 dB preamplifier (PA133_MR, 100 kHz to 15 MHz operating band down to -3dB from max amplification, MR-compatible, Sonic Concepts, WA, USA) and used to passively acquire acoustic emissions during sonication. For *ex vivo* experiments, the device was positioned horizontally into the tank, at the vicinity of the heart, and oriented toward the sonicated region. *In vivo*, the device was positioned on the skin of the animal, using vertical and horizontal positions measured from MR images acquired prior to sonication. Time-frequency maps were derived from the backscattered temporal signal using a customized spectrogram function (Hamming window, 98% overlap, 1024-point Fast Fourier Transform in MATLAB® 2015b, Mathworks, MA, USA). Harmonics of the HIFU frequency were filtered out in order to account only for broadband noise. Total broadband energy within the 1.2-25 MHz frequency band was computed as a metric of inertial cavitation[24,25] and compared to the same computation performed in the absence of sonication to determine noise level. An unpaired two-tailed Student's t-test was used to determine if the broadband energy measured was significantly higher ($p < 0.05$) than the noise for each

pressure. Results obtained from 2189 sonications *ex vivo* and 240 sonications *in vivo* targeting the left ventricle in both cases can be seen in figure 7.

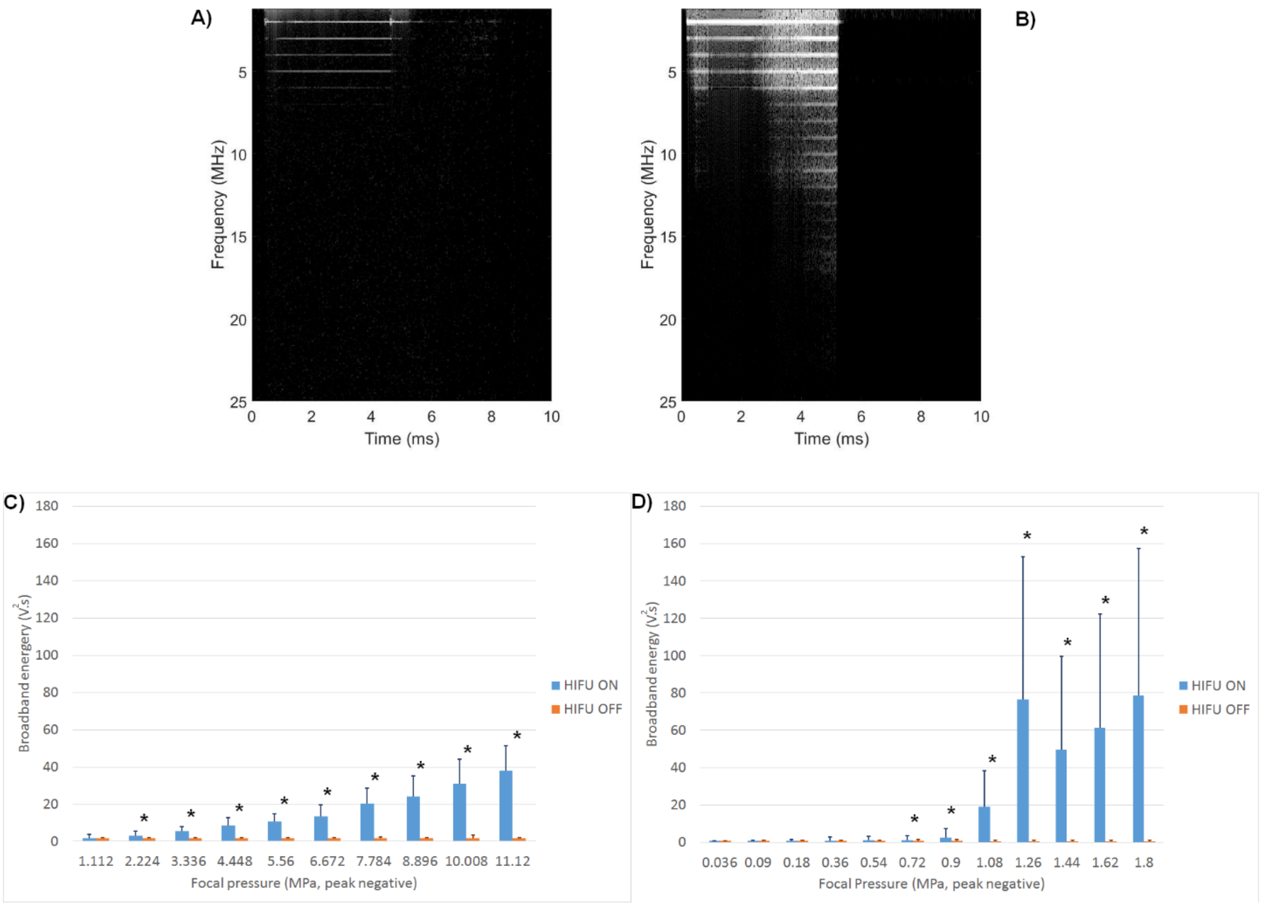

*Figure 7. Examples of spectrograms in the 1.2MHz-25MHz frequency range obtained in vivo with no significant inertial cavitation (A) and with significant inertial cavitation (B). Histograms show values of broadband backscattered acoustic energy measured during both ex vivo (C, N=2189) and in vivo (D, N=240) experiments. * denotes a p-value < 0.05 (unpaired two-tailed Student's t-test) between acoustic sonication and noise level and therefore significant detection of inertial cavitation.*
